# Simple Complexity: Incorporating Bioinspired Delivery Machinery within Self-Assembled Peptide Biogels

**DOI:** 10.3390/gels9030199

**Published:** 2023-03-06

**Authors:** Rui Li, Qing-Ling Zhou, Min-Rui Tai, Kathryn Ashton-Mourney, Mathew I. Harty, Aaqil Rifai, Clare L. Parish, David R. Nisbet, Sai-Yi Zhong, Richard J. Williams

**Affiliations:** 1 College of Food Science and Technology, Guangdong Ocean University, Zhanjiang 524008, China; 2 Collaborative Innovation Center of Seafood Deep Processing, Dalian Polytechnic University, Dalian 116034, China; 3 IMPACT, School of Medicine, Deakin University, Geelong, VIC 3217, Australia; 4 The Florey Institute of Neuroscience and Mental Health, The University of Melbourne, Melbourne, VIC 3052, Australia; 5 The Graeme Clark Institute, The University of Melbourne, Melbourne, VIC 3010, Australia; 6 Department of Biomedical Engineering, Faculty of Engineering and Information Technology, The University of Melbourne, Melbourne, VIC 3010, Australia; 7 Melbourne Medical School, Faculty of Medicine, Dentistry and Health Science, The University of Melbourne, Melbourne, VIC 3010, Australia

**Keywords:** self-assembling peptides, gene delivery, drug delivery, smart delivery platform

## Abstract

Bioinspired self-assembly is a bottom-up strategy enabling biologically sophisticated nanostructured biogels that can mimic natural tissue. Self-assembling peptides (SAPs), carefully designed, form signal-rich supramolecular nanostructures that intertwine to form a hydrogel material that can be used for a range of cell and tissue engineering scaffolds. Using the tools of nature, they are a versatile framework for the supply and presentation of important biological factors. Recent developments have shown promise for many applications such as therapeutic gene, drug and cell delivery and yet are stable enough for large-scale tissue engineering. This is due to their excellent programmability—features can be incorporated for innate biocompatibility, biodegradability, synthetic feasibility, biological functionality and responsiveness to external stimuli. SAPs can be used independently or combined with other (macro)molecules to recapitulate surprisingly complex biological functions in a simple framework. It is easy to accomplish localized delivery, since they can be injected and can deliver targeted and sustained effects. In this review, we discuss the categories of SAPs, applications for gene and drug delivery, and their inherent design challenges. We highlight selected applications from the literature and make suggestions to advance the field with SAPs as a simple, yet smart delivery platform for emerging BioMedTech applications.

## 1. Introduction

**Figure 1:**
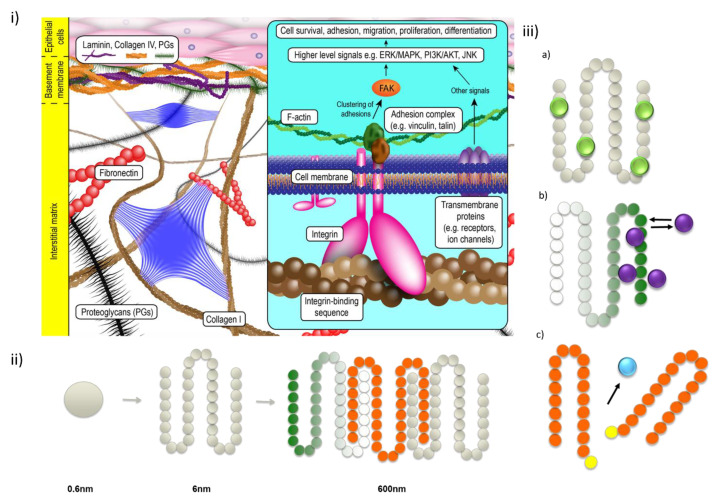
Self-assembling peptides as mimics for the natural cellular environment. (**i**) Cells exist within the extracellular matrix, an information-rich support scaffold. Key to this activity is the formation of recognition sites between the cells and peptide sequences within the fibrous structures and proteins of the Extracellular Matrix (ECM). (**ii**) Self-assembling peptide structures are excellent synthetic analogues for the ECM. Simple subunits are synthetically coupled to yield chains, which then interact with self-recognition to form structures of similar length scales to the ECM; with careful design, these SAPs can also present the integrins required by nature and include regions that bind and release drugs and bioactive molecules. (**iii**) Drugs can be incorporated to and be delivered from these SAPs by adding functionality to the amino acid sequence, such as (**a**) covalent (permanent) presentation, (**b**) electrostatic (reversible) attraction or (**c**) biocatalytic activity.

Peptides, short amino acid sequences, are useful tools to create therapeutics and biomaterials [1]. Peptide-based molecules, carefully designed to spontaneously self-assemble, can form structurally rich hierarchical supramolecular constructs. The final form of these is dependent on molecular composition, assembly kinetics and the environment (solvent, pH, temperature, ionic strength and co-assembly molecules, etc.); a key toolbox that enables the rational inclusion of specific biochemical signatures [2]. Peptides are ideal candidates for self-assembly, as the sequence of amino acids in the parent proteins molecules determines the ordering of structures in nature. These amino acids are chemically rich, and when incorporated into short sequences, enable intermolecular interactions such as van der Waals and electrostatic forces, hydrogen bonding and π-π stacking while avoiding the folding and intramolecular interactions present in longer polypeptides. These means the short (e.g., 1–10 residue) peptides can contribute their interactions primarily for the arrangement of molecular components into hierarchically ordered nanostructures, such as tubules, ribbons, spherical or cylindrical micelles, vesicles and hydrogels [3], all of which are promising materials for biomedical applications [4]. Importantly, the programmed and synthetically straightforward peptides enable a bottom-up strategy to create nanosized biomaterials to create functional materials for many applications, such as drug delivery and tissue engineering (Figure 1) [5,6].

These minimalist sequences are known as self-assembled (or assembling) peptides (SAPs). SAP materials are ideal candidates for BioMedTech applications, as they are inherently biodegradable with predictable degradation products from the activity of, for example, proteases [7]. They are versatile, as altering amino acid sequences enables chemical diversity that can be tailored toward specific biological processes, such as inclusion of epitopes for cell attachment and compounds for immunomodulation [8]. These features have been employed to move the simple systems forward by modifying them to incorporate more complex drug delivery machinery, such as specific and long-term release. Recently, this growing field has advanced from approaches for the simple solubilization of hydrophobic drugs to ‘smart’ vectors that respond to a disease biomarker, can support cell and gene delivery, and present vaccines [9]. In this review, we will discuss some of these advances in detail. 

## 2. Advantages of SAPs as Delivery Tools

In order to deliver drugs to specific tissues, several challenges must be overcome. These drugs must be presented at a specific concentration, time and place; typically determined by the biological needs of the therapeutic or regenerative process. This presentation must ensure that the therapeutic payload is both protected from the biological environment that may degrade it, administered in a dose-dependent, safe manner [10] to minimize off-target effects, and be co-located to the site of therapeutic need [11]. Numerous strategies have been developed to achieve this, from simple injection [12], through to biologically stimulated release [13]. This typically entails loading drugs onto (nano)particles [14], encapsulating in vesicles [15], reversibly attaching to polymers [16], or using bulk hydrogels to immobilize the drug via steric hinderance [17]. Hydrogels are typically considered to be networks consisting of a structure that encapsulates a significant amount of water [18]. This hydrated nature enables water-soluble fractions to be loaded into the system or added to structures that can be embedded. Such hydrogels are naturally derived (hyaluronic acid, chitosan, gelatin, cellulose etc.) or synthetically produced (polyethylene glycol, polyacrylamide, polycaprolactone etc.) [19].

An emerging category of hydrogelators are short peptides that assemble into nano-micro-macroscale hierarchical materials [20]. There are several benefits of peptide self-assembly to form the core of a functional biogel. Compared to macromolecules, SAPs are small, can be routinely synthesized and are easily purified immediately after synthesis. Solid-phase peptide synthesis is the stepwise addition of amino acids from a solid support, and this stepwise process allows the amino acid sequence to be precisely controlled, enabling simple molecular design (in even a basic chemistry lab), which opens the field up to rapid development of cross-disciplinary work to non-specialists in the field [7]. Beyond the simple one or two component systems, there are opportunities to generate modular, combinatorial libraries from synchronous co-assembly of various peptides tuned towards specific biological requirements and therapies [21,22]. Compared to the variety of polymeric and other soft materials employed by researchers, the simple peptide assembly approach becomes even more appealing, as it provides an easy way to include key chemical functional groups and moieties, including nonpeptidic groups (e.g., drugs, polymers, lipids) into its structures that enable facile modification of the functional spine [1]. 

Stable, highly hydrated, interconnected hierarchy of structures, SAP delivery systems offer desirable characteristics for high drug loading, low drug leakage, sustained delivery, and high permeability to reach the target cells [9]. Furthermore, amphiphilic peptide systems have been shown to encapsulate hydrophobic compounds within their structure, giving rise to a encapsulated reservoir that can be positioned in space, enabling the precise control of drug release, protecting the drug from exposure to degradation factors and decreasing risk of tissues being exposed to toxic drugs [23]. For drug delivery purposes, chemical modifications of SAPs is a highly promising approach for improving their enzymatic stability and/or cell membrane penetration ability, as well as reducing immunogenicity [24]. 

## 3. Classification of Self-Assembling Peptides

### 3.1. Amphiphilic Linear Peptides

Amphiphilic peptides have both hydrophobic and hydrophilic faces. Significant success has been achieved with peptide amphiphiles (PAs). These are a peptide-alkyl chain with four basic structural factors: a hydrophobic portion, a short peptide sequence able to form intermolecular hydrogen bonds and promote β-sheet formation, a charged amino acid sequence to balance aqueous solubility and a domain for the inclusion of bioactive signals [25]. Therefore, PAs are likely to assemble into β-sheet segments, giving rise to highly one-dimensional nanostructures that underpin hydrogel networks [26]. However, some PAs have dynamic secondary structures, for example, changing from β-sheet to α-helix with either a temperature or pH change [27]. PA morphologies vary according to different molecular components and have been reported to produce shapes such as scroll-like cochleates, flat nanobelts, cylinders, twisted ribbons and micelles [28]. Early in the field of PAs, Zhang et al. discovered a SAP sequence, EAK16, in Zuotin (a Z-DNA binding yeast peptide) in 1992 and subsequently designed RADA16 [29,30], with both being general peptides without any active motifs. Stupp et al. also significantly contributed to the field, developing several peptide sequences [28]. Sangji et al. designed a class of PAs using a similar design concept, but with the main part of the peptide composed of commutative hydrophobic and hydrophilic amino acids and the terminal consisting of charged amino acids to form multidomain peptides [31]. On the other hand, surfactant-like peptides, such as Ac-A_6_KNH_2_ and KA_6_-NH_2_, were also designed by Zhang’s group. These have a nonpolar tail composed of hydrophobic amino acids and a hydrophilic head consisting of charged amino acids and are capable of assembling in water to form nanostructures including tubes, vesicles and micelles [32].

### 3.2. Cyclic Peptides

Cyclic peptides are able to form rigid nanotubes via hydrogen bonding and allow the molecules to present a variety of orientations that are not available in linear peptides [33]. They have been shown to have several favorable properties, such as strong binding affinity, target selectivity and low toxicity, which make them an attractive modality for the development of therapeutics [9] and nanoparticles [10]. The vast majority of clinically-approved cyclic peptides are derived from natural products [34]. Specifically, cyclic dipeptides (CDPs) are a category of hormone-like molecules that are naturally occurring in organisms from bacteria to humans [35]. CDPs are able to form nanostructures spontaneously under much milder, aqueous conditions [36]. A recent report indicated that CDPs preferentially assemble into ordered hierarchical structures compared to their linear corresponding forms due to the absence of free amino or carboxylic groups [37]. From a molecular design perspective, the rigidity initiated from the cyclic structures and the strong hydrogen bonding ability facilitate assembly of CDPs into more complex architectures. In mammals, CDPs have been shown to interact with glial cells, thereby having a potential to treat neurodegenerative diseases. Furthermore, CDPs are well known for their ability to cross the blood-brain barrier (BBB) and are able to decrease inflammation and induce a protective state in neurons [38]. 

### 3.3. N-Terminally Protected Peptides

The N-terminal residue of the peptide chain can be modified with a capping group. This enables a hydrophobic or aromatic group to be introduced and allows shorter fragments to self-assemble. For example, Fluorenylmethoxycarbonyl (Fmoc) Fmoc-protected peptides are a class of minimalist peptides, with short amino acid sequences of 1–10 residues and can form hydrogels underpinned by nanofibers under physiological conditions [11,39]. For these systems, supramolecular assembly is governed by a combination of aromatic stacking interactions and the tendency of the peptides to form a β-sheet-type hydrogen bonding arrangement [40]. This assembly motif can drive self-assembly of otherwise soluble short peptide sequences into nanofibrils through π-π stacking of the Fmoc aromatic domains [41,42,43,44]. Smith et al. suggested the self-assembly mechanism that encourages the derivatives to form β-sheets between the peptide sequence, resulting in the presence of the amino acid side chains on the surface of the nanofibrils through π-β assembly [45]. Bundles of these nanofibers then interact through supramolecular ordering to induce the formation of a highly-interweaved nanofibrous network that intertwines to microscale scaffold and forms a macroscale hydrogel [41]. Importantly, the amino acid side chains of the peptide sequences are presented on the surface of the nanofibers [21]. This can provide surface biofunctionalization as a direct consequence of the assembly mechanism, meaning the C-terminal residues are available for interaction with cell surface receptors [45,46]. Peptide sequences are presented on the surface of proteins, which are then recognized by cells. Most commonly utilized are the synergistic peptide epitopes RGD and PHSRN derived from fibronectin that engage the integrin receptor for cell adhesion, while IKVAV and YIGSRF are pentapeptide epitopes of the basement membrane protein laminin [47]. Several peptides of this class have been developed, utilizing aromatic stability (such as Fmoc-FF/Fmoc-RDG blends [48], Fmoc-FRGDF [20,21]), pka modification (Fmoc-D(D)IKVAV(D/K) [49,50], Fmoc-DYIGSRF [51]) and direct inclusion (Fmoc-PHSRN [52]). All of these are able to assemble to form transparent nanohydrogels and have been employed in as a tissue-mimicking platform for different applications, such as cell culture [20,21,53], localized virus vector gene delivery [50] and central nervous system repair [51]. 

### 3.4. Hybrid Peptide Assemblies

Triggering the assembly of SAPs in the presence of other functional molecules further expands the library of structural and functional diversity. Carefully selected, proteins and polymers can become part of the hydrogel, adding richness and complexity to target the requirements of a greater number of applications [54]. SAPs have been co-assembled with self-organising molecules such as lipids, nucleic acids (including DNA and RNA), polysaccharides, proteoglycans, supramolecular organic molecules, and metal ions. Many reports highlight the utility of peptide/lipid materials, noting the combined advantages of formula stability and biocompatibility in vitro and in vivo [55]. Recently, Saher et al. engineered a peptide/lipid scaffold to deliver oligonucleotides with a demonstrable improvement of in vitro and in vivo transfection [56]. Furthermore, enhanced materials have been developed for gene delivery with the engineering of ionizable lipids and lipid-like materials [55]. Kim et al. designed a dual-bioactive copolymer consisting of oligonucleotides and peptides, acting as a two-polymer complementary system. This assembles into various 1- and 2-dimensional structures, such as nanofibers, ribbons, and sheets through controllable amino acid interactions, and can undergo protease-induced fiber-to-sheet shape transformations [57]. Li et al. showed co-assembly of a SAP with a polyanion polysaccharide, fucoidan, and a developmentally important proteoglycan, versican, which decorated the final assembled nanostructure and effectively enabled the material to support the lineage correct development of muscle [20] and neural progenitor cells [22]. These simple, elegant, yet complex interactions also enable the microscale ordering of peptide fibrils and subsequent mechanical properties of the resultant hydrogel to be easily and effectively managed without disruption to the desirable nanofibrillar structure of the assembly [11]. This property has enabled SAP hydrogels that are mechanically weak to be combined with robust polymers to create biolinks that are stable enough to tolerate the shear profile and still enable the fine resolution of constructs for biofabrication [4,58,59,60].

## 4. Delivery of Bioactive from SAPs

Due to their versatility, SAPs have been used as a platform technology to stabilize and control delivery of biological compounds that are expensive, complex or liable to degradation. Table 1 summarizes selected reported SAPs and how they have been used for a variety of application-specific therapeutic delivery.

### 4.1. Anticancer Drug Delivery

Cancers are one of the most fatal diseases in the world; increasing numbers of new cases each year mean new, cheaper and safer therapies are urgently required [96]. Nanotechnology-based drug-delivery systems are widely expected to bring new hope for cancer therapy by overcoming the problems of the main anticancer treatment methods in clinical use [61]. Peptide-based nanomaterials have been used for the delivery of chemotherapy compounds for a range of diseases and tissues. One vital design rule is the controlled release of therapeutic agents over a certain period of time to target the tumor and limit off-target effects [13]. Spatial accuracy is crucial in drug delivery, especially to increase the efficacy and reduce the side effects of antitumor drugs. The non-covalent and tissue-specific tuning of the assembly process make injectable SAP hydrogels formed under physiological conditions excellent carriers for compound delivery [3]. SAP systems have been successfully applied to deliver a number of anticancer therapeutic agents, including Doxorubicin (DOX) and paclitaxel (PTX) [13,15,61,62,63,64,65,66,67,97]. For example, an SAP hydrogel was used for the co-delivery of DOX and curcumin for effective combination therapy and targeted treatment of tumors situated in the neck and head [67]. Zhang et al. fabricated a cyclic peptide-based nanotube, which was modified using polyethylene glycol coating to bind DOX [69]. An amphiphilic peptide dendrimer (AmPD), which assembled into nano-carriers for effective encapsulation DOX, improved the intracellular uptake and accumulation of DOX in drug-resistant breast cancer cells and improved permeation in 3D multicellular tumor spheroids compared with free DOX [61]. SAP hydrogels can be employed as model systems to assess the effectiveness of drugs in a more tissue mimetic 3D environment than the traditional 2D systems, enabling the modeling of metastasis and the utility of embedded drugs [18], as well as studying the chemotaxis of antitumor T cells in response to cytokine gradients [98]. Bai et al. developed an amphiphilic SAP allowing for the encapsulation of DOX in a neutral medium. The DOX-encapsulating peptide nanoparticles swelled and burst in an acidic microenvironment, releasing DOX in tumors, resulting in significant antitumor effects in vivo [63]. 

The inherent ability of SAP to mimic the epitopes of ECM proteins has been utilized to bind to overexpressed proteins on vascular endothelial cells and tumor cells [63,68]. This can then stimulate endocytosis and help the cargo to penetrate cancer cells [3]. The anticancer agents are then released directly adjacent to the tumor via cleavage by highly expressed local factors, such as matrix metalloproteinase-2, which accelerate the spatially confined biodegradation of the gel [99]. Boron neutron capture therapy is an emerging tumor selective therapy; Michiue et al. used self-assembling A6K peptide nanotubes as boron carriers. A6K nanotube delivery improved two major limitations of boron: the absence of intracellular transduction and non-specific drug delivery to tumor tissue. As a result of the targeted delivery, 10 times higher intracellular boron concentration than the current therapy was achieved [70].

### 4.2. Cardiovascular Delivery

Heart failure remains one of the leading causes of death worldwide, most commonly developing after myocardial infarction (MI) [100]. Cells lost during MI are not replaced, and with limited regenerative capacity (≤1% cardiomyocytes replaced annually), the tissue lost is replaced by a non-contractile fibrotic scar [101]. Currently, heart transplantation remains the only curative treatment. However, transplantation is not always possible owing to donor shortages and patient ineligibility. Therefore, there is an increasing need for the development of cell-based therapies to promote regeneration of the heart after MI [73]. Major challenges of cellular therapy for cardiac regeneration are poor transplantation efficiency, survival of delivered cells and lack of a myocardial niche environment that recapitulates the biochemical and mechanical cues of the developing myocardium. In addition to this, implanted and/or residual cells require additional factors to promote survival, growth and differentiation to support regeneration. Therefore, SAPs have emerged as promising materials to address these shortcomings and enhance cell therapy for cardiac repair. 

SAPs have numerous inherent advantages for delivery to the heart; in particular, their general biocompatible properties and the ability to form stable and injectable nanofibrous hydrogels that support cell transplantation. SAP-based protein delivery has been used for the myocardium to enhance cell therapy, progenitor/stem cells delivery, endothelial cells delivery and myocytes delivery [102]. Burgess et al. explored the potential of using an injectable, RGDSP-functionalized SAP—FEFEFKFK—hydrogel as a scaffold for the delivery and retention of rat cardiac progenitor cells (CPCs) into the heart. Injection of the hydrogel on its own or loaded with CPCs into the rat after injury resulted in a significant decline in myocardial damage and left ventricular dilation [73]. 

In order to achieve controlled delivery of proteins using SAPs, it is crucial that the scaffold components and pores match the particular size and composition of the protein molecules being delivered [103], requiring further studies to optimize self-assembling systems to achieve desired release. Of relevance, Koutsopoulos et al. examined release kinetics of various proteins with different physicochemical properties from a (RADA)_4_ gel, and found that varying peptide nanofiber density could be an efficient approach to control protein release [104]. Furthermore, the net or spatial distribution of charges, the hydrophobicity of the amino acids constituting the peptide sequences, and the lipophilicity/hydrophobicity and surface chargers of the therapeutic protein all influence release kinetics from SAP nanofibers. 

The intrinsic ability of SAPs to promote endothelial progenitor cell recruitment within its microenvironment has inspired further work specifically targeting vasculogenesis, angiogenesis and arteriogenesis following MI. RADA 16-II nanofibers were employed to deliver vascular endothelial growth factor (VEGF), resulting in sustained presentation over >14 days in the myocardium and resulted in improved angiogenesis, arteriogenesis and cardiac function at 28 days post-MI in rats [74]. Interestingly, results also showed that the cell adhesion molecule β2-integrin expression was upregulated in the myocardium of these animals. Success resulted in duplication of the study in pigs, a critical prerequisite in the pipeline to clinical translation. In a similar study, Guo et al. developed a bioactive peptide by attaching the heparin-binding domain LRKKLGKA to RADA 16-II peptides, thus promoting the binding of VEGF to the scaffold and slowing release after injection into the myocardium [75]. The benefits were VEGF retention for 28 days in a rat model of MI. Although VEGF delivery via unmodified RADA 16-II nanofibers improved capillary vessel density, increased ventricular function, and reduced apoptosis and scar formation, the adoption of the heparin-binding domain caused dramatically stronger results in all aspects, proving the merits of sustained local delivery. 

### 4.3. Bone Delivery

The delivery of osteoinductive SAPs have gained attention and shown promise for accelerating bone repair [105]. A void-filling network of SPG-178 nanofibers yielded hydrogels shown to have some inherent osteo-inductive properties [106,107]. PA-based biomaterials delivering low doses of recombinant BMP-2 (a bone growth factor), have successfully healed bone defects [78]. This was improved with a modified SAP with higher affinity that successfully slowed BMP-2 release, with in vitro experiments demonstrating that the stored and released BMP-2 retained its bioactivity but significantly increased its stability [76]. 

While these studies adopted relatively short peptide sequences (16 and 13 amino acids long, respectively), longer peptide chains can also be used for BMP delivery to enhance bone regeneration. For example, Poly(VPAVG)_220_ is a thermal-responsive elastin-like polymer with the sequence VPAVG repeated 220 times. This resulted in self-assembled spherical nanoparticles that were capable of encapsulating and sustaining BMP-2 delivery [79]. 

Bone remodeling has also been targeted by an SAP to deliver RANKL, a factor contributing to bone turnover. Kaipatur, et al. showed a (RADA)4 hydrogel with RANKL could induce osteoclastogenesis in vitro [77]—with proposed benefits for controlling tooth movement in orthodontic procedures. 

### 4.4. Preipheral Nervous System Delivery

Damage to the peripheral nervous system, most notably spinal cord injury (SCI) has thus far evaded satisfactory therapeutics in clinical trials [108]. Whilst biomaterials have been heavily investigated for their potential in spinal cord repair, a significant challenge associated with the use of preformed polymeric scaffolds has been the space requirement—requiring removal of scar tissue from the lesion site to accommodate the implantation. SAPs alleviate this challenge with their ability to fill tissue voids and self-assemble in situ, whilst also modulating local inflammation to minimize scar formation [109]. Whilst SAPs have demonstrated benefit in their own right in facilitating repair in SCI models, similar to other tissues, they promise superior drug and/or protein delivery kinetics at the lesion site to modulate local inflammation, enhance angiogenesis and neuritogenesis as well as provide neuroprotection to minimize injury and/or promote spinal cord repair [80]. 

Taxol-loaded SAPs have been studied, an antitumor drug, which is also recognized for its ability to promote neurite regeneration via stabilization of microtubules. Taxol released from the SAP prolonged its activity to promote neurite extension, reduce glial scarring and consequently result in enhanced functional recovery after SCI in canines without notable cytotoxicity [110]. In a confirming study, rats with SCI treated with SAP/Taxol similarly exhibited neurite preservation, smaller lesion sizes and decreased inflammation. [80]. Work by Luo et al. saw the fabrication of a hybrid injectable hydrogel (Fmoc-protected chitosan and Fmoc-protected laminin) that encapsulated curcumin to sustain release [81]. This functionalized hydrogel, well tolerated due to the biomimetic properties matching rat spinal cord, also modulated local inflammation and supported the infiltration of endogenous stem cells to participate in remyelination of regenerating nerves, leading to improvements in hindlimb function [81]. 

In another study, Lindsey et al. examined the feasibility of using MAX8 as a delivery system for nerve growth factor (NGF) and brain-derived neurotrophic factor (BDNF); two key neurotrophic growth factors currently used in experimental treatments of spinal cord injuries. Importantly, the encapsulation of NGF and BDNF within MAX8 did not negatively impact gel formation, nor the shear thinning property of gels result in immediate growth factor release [84]. Meanwhile, Hassannejad et al. used a PA functionalized with the laminin epitope, IKVAV, to provide sustained release of BDNF. Injection of the BDNF-loaded IKVAV PA hydrogel resulted in considerable axon preservation and astrogliosis reduction at 6 weeks post-injury [83]. 

In SCI, the formation of the glial scar is considered one of the greatest barriers to regeneration. The scar is composed of proteoglycans that impede axonal growth. Chondroitinase ABC (ChABC) is a depolymerizing lyase enzyme that cleaves a broad range of galactosaminoglycan substrates, including chondroitin sulfate proteoglycans, to reduce the gliotic scar [111]. However, its thermal instability hampers its clinical application [112]. Recently, Raspa et al. used two SAPs featuring different self-assembled nanostructures and different methods of drug loading to extend the activity of ChABC from its typical < 72 h to 42 days in vitro. Subsequent injections of the ChABC-loaded SAP hydrogels favored host neural regeneration and behavioral recovery in chronic SCI in rats. Therefore, SAP hydrogels showed great promise for their future therapeutic use in spinal cord repair—with key attributes in their minimally invasive delivery and vital capacity to sustain delivery of drugs, proteins and enzymes at the injury site targeted at driving regeneration [82]. 

### 4.5. Delivery to the Brain

There are additional challenges to successfully deliver a cargo to the brain compared to other tissues. A carrier must not only navigate through the body while eliciting minimal immunogenic response and avoiding off-target delivery, but also needs to achieve transport across the BBB, perhaps the most tightly regulated biological barrier in the human body [113]. 

Although still less exploited in the treatment of CNS disorders, a growing amount of work shows that SAPs have significant promise in delivery of compounds to the brain. Both polypeptide-bearing amphiphilic block-copolymers and PAs that self-assemble into peptide nanoparticles (NPs) [114], can be functionalized with ligands that target specific receptors expressed on brain endothelial cells or the nasal epithelium [115]. The widely explored RGD peptides have a selective affinity for the integrins overexpressed in the endothelial cells of tumor angiogenic vessels [85]. Recently, these cyclic-RGD (cRGD) peptides were also used for delivery of epirubicin for treatment of glioblastoma multiforme (GBM, a type of brain tumor) [86]. cRGD-epirubicin micelles showed a 12-fold increase in antitumor activity in an orthotopic GBM model compared to pristine micelles, and could also be employed to image the neovasculature in this model [116]. Kulhari et al. conjugated different RGD (cRGDfK peptide) nanoparticles delivery of camptothecin to GBM, which resulted in more efficient ROS generation, induction of apoptosis and improved control over cell migration [87]. Another class of peptide, cell penetrating peptides (CPPs), have also been reported for the delivery into the brain [117]. CPPs are short peptides (< 30 amino acids in length), that can pass through membranes without any interactions with specific receptors. Raucher et al. used a elastin-like polypeptide CPP to deliver a dominant negative Mastermind-like protein or DOX and demonstrated the effective killing of GBM cells [117,118]. 

In addition to the non-invasive capacity for drug delivery into the brain and across the BBB, SAPs have been investigated for their potential to promote repair following intracerebral delivery. Most relevant in stroke where, much like spinal cord injury, there is a lesion side, tissue void and glial scarring, here SAPs can impact regeneration directly because of their customized peptide-based properties, but also in their capabilities to deliver compounds (small molecules, proteins and drugs) in a targeted temporal and spatial manner to the infarct site. Most recently, Yaguchi et al. fabricated a cell-adhesive fiber forming peptide that mimics aspects of the ECM—forming micrometer-long supramolecular nanofibers that dispersed homogeneously in the hydrogel and could efficiently mediate the sustained release of vascular endothelial growth factor, to provide regeneration in a mouse stroke model. In a zebrafish brain injury model (severed optic tectum), a RADA16-SVVYGLR hydrogel promoted angiogenesis and invoked local neurogenesis resulting in functional recovery. Fmoc-protected SAPs matching the common brain ECM protein laminin have shown significant promise for the repair of stroke [22], cell delivery [119], and gene [120] and drug presentation [121]. This is achieved via the careful engineering of the binding profile of the SAP through inducing charge to the C-terminus. Fmoc-DIKVAV(K or D) gels were shown to be readily injectable, well tolerated, able to recruit host cells and deliver complex regenerative biomolecules with significant recovery of the brain’s architecture.

### 4.6. Ocular Delivery

Topical delivery of ocular therapeutics is commonly impaired by rapid tear clearance, poor corneal absorption and low bioavailability (less than 5%) [122,123]. Further drawbacks include blurred vision, difficulty in dosage loading amounts and immunogenic risks. Thus, there is an urgent need for developing new approaches to enhance the bioavailability and safety of the existing drugs. 

Considering the negative charges of the ocular mucosa, the rational design of cationic peptide-based molecular hydrogels is promising to prolong the ocular residence time of drugs, which simultaneously provides a mucoadhesive property via the electrostatic interaction with negatively charged mucin on the ocular surface. Li et al. used a cationic peptide (Nap-FFKK) for treating ocular disorders, exhibiting minimal cytotoxicity against human cornea and displaying excellent biocompatibility properties [92]. More recently, Nap-FFKKFKLKL/dexamethasone sodium phosphate (Dexp) hydrogel was observed to cause little damage to the retinal architecture and eyesight functions during the one month of follow-up after a single intravitreal injection. Dexp also had potent capacity to alleviate intraocular inflammation and retain retinal architecture, suggesting that this hydrogel may be a promising drug carrier system to treat various posterior disorders (i.e., uveitis) [93]. 

Karavasili et al. engineered two SAPs, (RADA)4 and (IEIK)3I, that both formed hydrogels at physiological conditions and were used as carriers for ocular delivery of timolol maleate (TM). They showed controlled release and transport of the drug through the cornea with (IEIK)3I showing a slower release than (RADA)4. Moreover, in vivo studies demonstrated that there was an extended reduction in intraocular pressure (up to 24 h) compared to the drug solution [89]. Recently, the (RADA)4 hydrogel was used for the ocular co-delivery of TM and brimonidine tartrate (BR). This achieved a 2.8-fold and 5.4-fold higher corneal permeability for TM and BR, respectively [90]. 

### 4.7. Pancreatic (Beta-Cell Replacement) Delivery

Transplantation of the insulin-producing β-cell islets is a promising treatment for Type 1 Diabetes. However, several factors such as immune-mediated transplant rejection and immunosuppressive-related beta-cell toxicity severely limit the long-term success [124,125]. Furthermore, transplanted islets lack an ECM. This significantly impairs their integrity and viability, also contributing to transplant failure [126]. To improve clinical islet transplant outcomes, encapsulation within biocompatible SAP hydrogels has shown a promising ability to protect islets from immune-mediated destruction, while providing an ECM-mimetic microenvironment to support beta-cell viability and insulin secretion.

Liu et al. functionalized the cationic SAP, KLD12 (KLDLKLDLKLDLR) with collagen IV and fibronectin mimicking motifs abundant in the native islet ECM to encapsulate islets and deliver sustained release of hepatocyte growth factor (HGF). In vivo, this hydrogel showed slow release of HGF, which inhibited the expression of pro-inflammatory cytokine pathway activation and T-lymphocyte infiltration in grafts and thereby improved β-cell survival and insulin secretion [94]. Khan et al. used a similar approach to encapsulate islets in a PA, including a glucagon-like peptide-1 (GLP-1) mimic sequence. This hydrogel displayed GLP-1-induced effects of increased β-cell function while protecting from cytokines [95]. Therefore, SAPs are a novel strategy with potential for a wide range of β-cell protective strategies to improve islet transplantation outcomes.

### 4.8. Immunogenic Delivery

Immunogenic stimulation arising from the development of vaccines has become a milestone in disease prevention and human health. Currently, whole pathogen vaccines are most often used clinically [127]. While these are effective at mounting an immune response, more minimal components, such as a short immunostimulating peptides, are able to modulate an adaptive immune response [128]. SAPs can be designed to have negligible immune response, as discussed above. However, they can also be designed to cause a potent response. Importantly, this can be completed without the addition of complementary adjuvants, instead relying on sequence and physicochemical characteristics of the SAP, as shown in Table 2 [9]. 

Yang et al. designed a set of reduction-responsive SAP precursors, which can be reduced by glutathione (GSH), forming hydrogels with different surface properties (E-gel, S-gel, and K-gel). When co-assembled with vaccines (E-vac, S-vac, and K-vac) prepared by mixing different precursors with antigens before GSH reduction, K-vacc promoted antibody production and antitumor immune responses more effectively than the other two vaccines. Therefore, co-assembly of SAPs and antigens could act as efficient vaccine delivery systems for improving antibody production, and different immune effects can be obtained by changing the surface properties of the SAPs [129]. An IKVAV containing peptide has been used as an intranasal self-assembled nanovaccine delivery tool, with positive results [47].

Recently, SAPs themselves have been reported to exhibit immune-stimulating effects, therefore, they could also be designed as vaccines [129]. Peptide-based vaccines contain minimal antigenic epitope(s) derived from malignant pathogen proteins. These well-defined peptide vaccines are safer than traditional whole-organism vaccines due to the removal of unnecessary biological materials that often cause allergic reactions and toxicity [130]. However, these peptide vaccines on their own have weak immunogens. Thus, an efficient delivery vector is required to improve immune responses without inducing any undesired negative effects. To this end, self-assembled amphiphilic peptides can be designed as adjuvant carriers [131]. Zhang et al. codelivered a model antigenic epitope and molecular adjuvant by forming SAP amphiphile micelles with a β-sheet structure. They found this could significantly alter vaccine immunogenicity both in vitro and in vivo, and could produce a desired cytokine profile in response to antigen restimulation [132]. In general, amphiphilic SAPs can incorporate the antigenic peptides, prevent peptides from degradation and extend antigenic peptide exposure to the immune system. Therefore, they could be a powerful tool for vaccine development [131]. 

**Table 2:** Applications of genetic and immunomodulatory delivery.

SAP	Delivered Molecule(s)	Secondary Structure	Physical Form	Factor Triggering Self-Assembly	Payload	Ref.
Gene Delivery						
FF	SiRNA	β-turn and antiparallel β-sheet	nanoparticle	electron attractive forces	breast cancer	[133]
GGGAAAKRK	SiRNA	β-sheet	aqueous dispersion	probe sonication	CNS pathologies	[134]
IKVAV	lentiviral	β-sheet	Hydrogel	pH-driven	CNS	[50]
KKALLHAALAHLLALAHHLLALLKKA	lentiviral	α-helical, coiled-coil	spherical particles	pH-driven	HCT116 cells	[135]
RRRR	pDNA	-	Ordered aggregates	aggregation-induced	HeLa cells, HepG2 cells, NIH 3T3 cells, 293T cells, stem cell R1	[136]
AAAAAAK	SiRNA	-	Nanotube	pH-driven	U87MG, U251MG, and T98G cells	[137]
**Vaccine delivery**						
GDFDFDYDX-ss-ERGD (X=E, S or K)	Tumor vaccine	β-sheet	Hydrogel	GSH	C57BL/mice	[129]

## 5. Novel Genetic Therapies

Increasingly, new therapies have focused on the delivery of genetic material that can elicit a response in the host cells (Table 2). Gene delivery is a targeted therapeutic strategy for renovation of diseased cells/tissues/organs or to impede disease progression [17]. Gene therapy has great potential to deal with the primary causes of incurable gene-related diseases, as well as help illustrate fundamental disease mechanisms in research [12]. During the processes of gene delivery, genes are introduced into host cells via vehicles. These vehicles can be either viral or nonviral and are particularly designed to preserve, secure and deliver genetic materials efficiently to a specific target cell colony. SAPs have been extensively developed for gene delivery applications [138,139] because they can simulate some features of the ECM of specific tissues [140], be engineered as identical single molecular substances and allow accurate management of characteristics, such as conformation, hydrophobicity and charge distance to incorporate a genetic payload on their surface [141].

A variety of delivery vehicles for nucleic acids depend on electrostatic interactions between positively charged groups and negatively charged nucleotide back bones. This approach can be extended to SAPs, which have demonstrated the ability to adsorb nucleotides by interactions with charged arginine or lysine residues [142]. The delivered molecules are typically siRNAs or short antisense oligonucleotides, but a recent report showed plasmid DNA could be stabilized by weaving the DNA over the positively charged surface of β-sheet-forming peptides [143]. However, the materials synthesized by this approach are easily degraded due to the surface adsorption of nucleotides. Although these materials may be suitable for transfection of cells in vitro or site-specific injection in vivo, this general approach may not work in circumstances where the cells or tissue are not directly contacted [134]. In addition, these peptides are often cationic, which means they can form electrostatic complexes with DNA, lowering the solubility of both charged species and inducing hydrophobic collapse and nanoparticle formation [144,145]. Once encapsulated into a synthetic nanoparticle, the therapeutic DNA is prevented from degradation and is more easily transported into the target cells. However, this process then needs to be reversible to facilitate release of the therapeutic gene. From this point of view, many DNA delivery formulations utilize subcellular processes to trigger DNA release from the vector [6]. 

SAPs have been used to deliver siRNAs for cancer treatment [133,146]. For example, Bozdoğan et al. encapsulated siRNA using diphenylalaninamide-based nanoparticles to silence human epidermal growth factor receptor-2 (HER2), a carcinogene playing a role in the development of some breast cancers. Functionalized diphenylalaninamide-based nanoparticles were prepared using layer-by-layer polyelectrolyte deposition. The SAP-based nanoparticles were capable of effectively delivering and releasing siRNA for silencing HER2 in breast cancer cells [133]. This study shows that as the anionic nature of siRNAs protected them from permeating into the cell membrane and escaping from endosomes, poly-L-lysine was used to modify the surface charge. Because siRNA can also affect other genes apart from the targeted gene, the use of SAP can help to prevent off-target uptake and undesirable toxicity [3]. Kostarelos et al. designed a surfactant-like peptide (palmitoyl-GGGAAAKRK) that was able to self-assemble into nanofibers, and demonstrated that complexes of peptide nanofiber/siRNA were taken up intracellularly, yet were spatially confined with an improved residence time of siRNA observed in the brain; thus, these complexes may be useful for use in central nervous system (CNS) pathologies [134]. 

Not every application is best suited for the use of a virus as a transfer vector. Novel systems that provide equally as effective gene transfer and minimal cytotoxicity are the main goals of these synthetic gene transfection systems, yet they have delivery and stability challenges which can be synergistically overcome with SAPs [136]. Liang et al. designed a small-molecule gene vector with aggregation-induced emission properties by capping a peptide containing four arginine residues with tetraphenylethene and a lipophilic tail. This vector can self-assemble with plasmid DNA to form nanofibers in solutions with low cytotoxicity and high stability. Transfection efficiency was also high, with the complexes able to transfect many cell lines, including stem cells [136]. A6K comprises six alanine residues and one lysine and has been reported as an siRNA delivery tool [137]. Hwang et al. designed novel synergistic nanocomplexes for effective transfection by incorporating the fusion of a nuclear localization signal and cell penetrating peptides with calcium phosphate. Fusion peptides were able to package large plasmid DNAs into nanocomplexes spontaneously and efficiently; thus, this strategy may be an effective and safe gene vector to treat various genetic diseases [147]. 

Viral vector gene delivery is a promising technique for the therapeutic treatments of proteins to damaged tissues for the enhancement of regeneration outcomes in various disease circumstances, including brain and spinal cord injury, as well as autoimmune diseases. Viral vector gene delivery is different to nonviral-based formulas, due to its significant numbers of empty capsids and limited size of therapeutic gene delivered based on the viral capsid capacity. However, SAPs can be used for viral vector gene delivery as well. For clinical applications in gene therapy, transduction protocols include the polypeptide CH-296, a sequence of the human fibronectin protein that enhances transduction by promoting the co-localization of viruses and cells and making the target cell more available to the carrier. Identification of new additives that are easy to manipulate and able to improve the infectivity of a wide spectrum of lentiviral vector (LV) phenotypes is therefore required [135]. Rodriguez et al. employed an aromatic peptide, Fmoc-DDIKVAVK, designed for localized viral vector gene delivery in vivo [50]. Recently, clinical trials using LVs obtained success, although the efficiency can be further improved by increasing transduction levels of target cells and the safety optimized by reducing the number of viral vectors [135]. However, virus vectors are costly and can cause inflammation and immunogenicity; thus, non-viral carriers may be more appropriate to develop for a wide range of gene therapy applications. 

## 6. Conclusions and Future Perspectives

SAP nanostructures have shown promising results in the delivery of therapeutics to a range of different tissues and cells. The predominant superiorities of SAPs are due to their simplicity and the relatively easy production and functionalization approaches [113]. However, despite several decades of research, peptide self-assembly is still poorly understood, and several challenges remain in the engineering, functionalization and development of peptide nano-assemblies as drug delivery vectors [9]. 

Characterization of SAP hydrogels is quite limited. Commonly used techniques such as dynamic light scattering and transmission electron microscopy give only partial information. Newer methods such as nanoparticle tracking analysis and tunable resistive pulse sensing have potential; however, the ability to determine more than just the average surface functional group density is incomplete [148,149].

With regard to SAPs as gene-delivery vectors, the peptide engineering library and therapeutic DNA formulations provide a toolbox of options. However, peptide-based gene therapy has several challenges that still need to be overcome, including unacceptable aggregation, pH stability, size control, and chemical and enzymatic stability [2]. New DNA formulations could be superior with respect to safety and effectiveness, but remain mostly unexplored under the setting of peptide-based gene delivery. However, ideas of bottom-up assembly between peptides and DNA could be effective. [6]. Future peptide gene-delivery nanocarriers need to realize reducing peptide toxicity, promoting site-specific degradability and target DNA release. Peptide degradability and toxicity are entwined, and utilizing enzymes within target cells to facilitate enzyme-specific degradation for DNA cargo release would minimize toxicity rapidly. 

Despite the challenges listed above, regarding brain drug delivery, peptide nanocarriers are perfectly positioned to achieve the successful delivery of treatments to the brain. The toolbox of peptide building blocks affords opportunities of design and tuning the abundant supramolecular interactions. Improved peptide nanomaterial synthesis approaches will, in turn, afford researchers new probes for recognizing novel routes and concepts for the delivery of therapies to the brain. The diversity of both material properties and experimental approaches makes peptide nanomaterials an extremely promising platform for use as delivery vehicles [113]. 

SAP-based nanomaterials are likely to play a valuable role in the delivery and targeting of future drug and vaccines. Further research is needed before this growing field can be translated to clinical application. However, future peptide delivery systems will likely transform into natural-synthetic hybrids that meet non-toxic functional demands and safety and efficacy purposes. For successful clinical application, peptide nanostructures must have improved stability within environments of varying pH, and the relationship between the morphology, physicochemical and biological activity must be exploited in more detail. Future work needs to focus on rational systematic design, which will help to understand peptide self-assembly thoroughly and allow specific and controlled development. In this way, SAPs could form the basis for a broad range of therapeutics and be a significant step forward in the treatment of numerous conditions. 

## Figures and Tables

**Table 1:** Applications of SAP-based delivery tools.

SAP	Delivered Molecule(s)	Secondary Structure	Physical Form	Factor Triggering Self-Assembly	Payload	Ref.
Anticancer drug delivery						
AmPDKK2/AmPDKK_2_K_4_	doxorubicin	β-sheet	nanoparticle	hydrophobic and hydrophilic interactions	breast cancer MCF-7R cells	[61]
GGVVVRGDR	paclitaxel	β-sheet	Hydrogel	Ion	U87 cancer cells	[13]
EVEALEKKVAALEC KVQALEKKVEALEHGW	Doxorubicin, apomorphine, rapamycin, tamoxifen, dexamethasone, paclitaxel	Worm-like Micelles	Spheres	hydrophobic interactions/geometric packing	-	[62]
LLLLLLKKKGRGDS	doxorubicin	β-sheet and random coil	nanoparticles	pH-driven	HepG2 cell	[63]
Adenine acetic acid-FFF	doxorubicin	Random structure	Hydrogel	pH-driven	4T1 cancer cells and tumor-bearing BALB/c mice	[64]
RADA16-I	Doxorubicin, curcumin	β-sheets	Hydrogel	Ion	Glioblastoma	[65]
Cbz-FF	Doxorubicin, curcumin	β-sheets	Hydrogel	pH-driven	-	[66]
RADA16-I	doxorubicin/curcumin	β-sheets	Hydrogel	Ion	head and neck cancer HSC-3 cells and HSC-3 tumor bearing SCID mice	[67]
RGD-PEG-SS-PTX	paclitaxel	-	Micelles	amphiphilic interactions	Human gastric carcinoma SGC7901 cells, mouse xenograft model of gastric tumor	[68]
PEG-QAEAQACA	doxorubicin	β-sheet	Nanotube	pH-driven	Human breast cancer MCF-7/ADR cells	[69]
AAAAAAK	Boron neutron	-	Nanotube	pH-driven	human U87 delta EGFR glioma cells	[70]
Chlorambucil -FFFK-cyclen	Chlorambucil	β-sheet	Hydrogel	heating-cooling	A549, Hela, MCF-7 cancer cells	[71]
GRVGPLGK	Doxorubicin/ paclitaxel/ curcumin	-	Hydrogel	ions	Hela, HT1080 cells	[72]
**Cardiovascular**						
RGDSP-FEFEFKFK	rat cardiac progenitor cells	anti-parallel β-sheets	Hydrogel	pH-driven	cardiac injury rats	[73]
RADA 16-II	vascular endothelial growth factor	β-sheets	Hydrogel	ion	myocardial infarction rat, myocardial infarction pig	[74]
LRKKLGKA-RADA 16-I	vascular endothelial growth factor	β-sheets	Hydrogel	ion	infarcted myocardium rats	[75]
**Musculoskeletal**						
RADA16-I	bone morphogenic protein-2	β-sheets	Hydrogel	ion	bone marrow stromal cell	[76]
RADA16-I	NF-kB ligand protein	β-sheets	Hydrogel	ion	Mouse macrophage cell line RAW 264.7 cells	[77]
Palmitoyl-AAAAGGGLRKKLGKA	Bone morphogenetic protein-2	β-sheets	Hydrogel	pH-driven	critical-size femoral defect rat	[78]
(VPAVG)220	bone morphogenetic protein-2	-	nanoparticles	temperature	C2C12 cells	[79]
**Peripheral Nervous System**						
RADA16-I-FGL	Taxol	β-sheets	Hydrogel	ion	Spin cord injury rats	[80]
Fmoc-IKVAV/Fmoc-chitosan	curcumin	β-sheets	Hydrogel	pH-driven	Laminectomy rats	[81]
FAQ (FAQRVPPGGGLDLKLDLKLDLK), CK (CGGLKLKLKLKLKLKGGC)	Chondroitinase ABC	β-sheet	Hydrogel	electrolytes and pH driven	Murine Neural Stem Cells, spin cord injury rats	[82]
IKVAV-PA	brain-derived neurotrophic factor	β-helices	Ion	ion	spinal cord injury rats	[83]
**Central Nervous System**						
MAX8	Nerve growth factor, brain-derived neurotrophic factor	β-hairpin	Hydrogel	ion	Growth factor delivery	[84]
Cyclic RGD linked polymeric micelles	(1,2-diaminocyclohexane) platinum (II)	-	Micelles	ion	orthotopic mouse model of U87MG human glioblastoma	[85]
Cyclic RGD linked polymeric micelles	epirubicin	-	Micelles	ion	glioblastoma	[86]
cRGDfK-conjugated γ-PGA	camptothecin	-	nanoparticles	hydrophobic and hydrophilic interactions	U87MG human glioblastoma cells	[87]
SynB1-VPGXG	doxorubicin	-	Conjugate	temperature	D54, GBM6, U251-MG glioblastoma cell lines	[88]
**Ocular Delivery**						
RADA 16-I/(IEIK)_3_I	timolol maleate	*β*-sheets	Hydrogel	ion	Rabbit eyes	[89]
RADA16-I	Timolol and Brimonidine	*β*-sheets	Hydrogel	ion	Glaucoma	[90]
ibuprofen– hydroxybenzoic acid –GFFY	ibuprofen	β-sheets	Hydrogel	Heat-cooling process	RAW264.7 macrophages, rabbit eyes, eye disorders	[91]
2-naphthylacetic acid (Nap)-FFKK	----	*β*-sheet	Hydrogel	ion	Rabbit eyes	[92]
Nap-KKFKLKL	dexamethasone sodium phosphate	α-helical	Hydrogel	noncovalent interaction	retinal architecture and eyesight functions of experimental autoimmune uveitis rat model	[93]
**Pancreatic Delivery**						
KLD12	Hepatocyte growth factor	β-sheet	Hydrogel	Ion	INS-1 beta-cell line	[94]
HSEDTFTSD	-	α-helical	Hydrogel	Ion	RINm5f cell line	[95]

## Abbreviations

Self-assembling peptides (SAPs); Extracellular matrix (ECM); Peptide amphiphiles (Pas); Cyclic dipeptides (CDPs); Blood-brain barrier (BBB); growth factor receptor-2 (HER2); Fluorenylmethoxycarbonyl (Fmoc); Central nervous system (CNS); Lentiviral vector (LV); Glutathione (GSH); Doxorubicin (DOX); Paclitaxel (PTX); mercaptoundecahydrododecaborate (BSH); Amphiphilic peptide dendrimer (AmPD); Myocardial infarction (MI); Cardiac progenitor cells (CPCs); Vascular endothelial growth factor (VEGF); Bone morphogenetic protein-2 (BMP-2); Spinal cord injury (SCI); Fmoc-protected chitosan (FC); Fmoc-protected laminin-derived peptide IKVAV (FI); Nerve growth factor (NGF); Brain-derived neurotrophic factor (BDNF); Chondroitinase ABC (ChABC); Chondroitin sulfate (CS); Dermatan sulfate (DS); Brimonidine tartrate (BR); Naphthylacetic acid (Nap); Peptide nanoparticles (NPs); Glioblastoma multiforme (GBM); cyclic-RGD (Crgd); Cell penetrating peptides (CPPs); Elastin-like polypeptide (ELP); Timolol maleate (TM); Dexamethasone sodiumphosphate (Dexp); Type 1 Diabetes Mellitus (T1DM); hepatocyte growth factor (HGF); glucagon-like peptide-1 (GLP-1); interleukin one-beta (IL-1β); nuclear factor kappa beta (NFκβ); dynamic light scattering (DLS); Transmission electron microscopy (TEM).

Abbreviation of amino acids: Arginine (R); Glycine (G); Aspartic acid (D); Phenylalanine (F); Glutamic acid (E); Alanine (A); Lysine (K); Isoleucine (I); Valine (V); Tyrosine (Y); Isoleucine (I); Serine (S); Proline (P); Histidine (H); Asparagine (N); Leucine (L).
